# Optimizing Gelatin Methacryloyl for Craniofacial Muscle Regeneration: Material Design and Application

**DOI:** 10.3390/gels11120945

**Published:** 2025-11-24

**Authors:** Mohammad B. Aljaber, Omar Alageel, David Y. S. Chau, Jonathan C. Knowles

**Affiliations:** 1 Department of Dental Health, College of Applied Medical Sciences, King Saud University, P.O. Box 10219, Riyadh 12372, Saudi Arabia; 2 Division of Biomaterials and Tissue Engineering, Eastman Dental Institute, University College London, Royal Free Hospital Campus, Rowland Hill Street, London NW3 2PF, UK

**Keywords:** hydrogel, gelatin methacryloyl, polymers, dentistry, tissue engineering, biomaterials

## Abstract

Gelatin methacryloyl (GelMA) hydrogels are widely used in tissue engineering because of their tunable mechanical and biological properties. However, many studies have arbitrarily selected key synthesis parameters, such as methacrylic anhydride (MA) concentration, (lithium phenyl-2 4 6-trimethyl-benzoyl phosphinate) LAP concentration, GelMA content, UV exposure time, and reaction duration, without clear justification. This study aimed to systematically optimize GelMA hydrogel fabrication and evaluate the mechanical and biological performances of the resulting hydrogels for craniofacial muscle tissue engineering. Hydrogels were synthesized following a standardized protocol, and the reaction progress was confirmed via proton nuclear magnetic resonance (^1^H-NMR). The swelling ratio, degradation behavior, compressive strength, and metabolic activity (AlamarBlue assay using C2C12 myoblasts) were assessed. Statistical analysis was performed using independent *t*-tests and one-way ANOVA with Tukey’s post hoc test (*p* < 0.05). The results showed that small variations in MA concentration and reaction time significantly affected the hydrogel properties. Higher GelMA concentrations (10–20%) enhanced the mechanical strength but reduced the biological activity. LAP ≥ 0.5% and prolonged UV exposure lowered metabolic activity, whereas 0.1% LAP with 1–2 min of UV exposure provided an optimal balance. These findings provide a reproducible framework for GelMA fabrication and establish a foundation for developing tailored biomaterials for muscle tissue engineering.

## 1. Introduction

Skeletal muscle regeneration, particularly in the craniofacial region, requires biomaterials that balance mechanical strength, controlled degradability, and high cytocompatibility. Gelatin methacryloyl (GelMA) is a photocrosslinkable derivative of gelatin that has gained significant attention in tissue engineering due to its ability to form stable hydrogels with tunable physicochemical and biological properties. Unlike unmodified gelatin, which relies on thermal gelation and exhibits limited mechanical stability, GelMA can be crosslinked under mild conditions using light exposure in the presence of photoinitiators, allowing precise control over stiffness, degradation rate, and porosity [1,2]. These tunable features enable GelMA to closely mimic the extracellular matrix (ECM) environment and support cell adhesion, proliferation, and differentiation [3,4]. Furthermore, the introduction of methacryloyl groups enhances batch-to-batch reproducibility and provides greater experimental control compared to natural gelatin [5]. In the context of skeletal muscle regeneration, GelMA has demonstrated excellent potential to promote myogenic differentiation and alignment of myoblasts, owing to its biocompatibility, adjustable elasticity, and suitability for micropatterning and 3D printing into anisotropic [2,4].

GelMA’s tunability arises from its modifiable components, such as polymer concentration, photoinitiator content, UV exposure, and synthesis conditions [6,7]. However, there is a lack of consensus on how to best optimize these factors for specific applications, such as myoblast growth and muscle regeneration [7].

GelMA hydrogels have been widely studied for tissue engineering. However, despite their growing popularity, many critical synthesis parameters, such as the concentration of methacrylic anhydride (MA) added during the reaction and the duration of the functionalization process, remain inconsistently reported and insufficiently studied in the literature. A survey of the literature reveals that the amount of MA added per 10 g of gelatin varies drastically across studies, ranging from 0.5 mL [1] to 20 mL [6,7]. Intermediate values such as 1, 2, 4, 6, 8, 10, and 12 mL have all been used [7,8,9,10,11,12,13,14], with no agreement on optimal conditions or justifications for their selection. In many studies, this parameter was not reported.

Similarly, the reported reaction times for GelMA synthesis range from 1 to 6 h. In the original protocol by Van Den Bulck [3], GelMA was allowed to react for 1 h, which was also reported by others [5,13,15,16,17,18,19]. However, many polymerization times have been reported, such as 2 h [20,21,22], 3 h [2,11,23,24], 4 h [9,25,26,27] and 5 h [28]. However, the impact of reaction time on hydrogel performance has rarely been studied in detail. These inconsistencies highlight a significant gap in the standardization of GelMA protocols.

**Figure 1:**
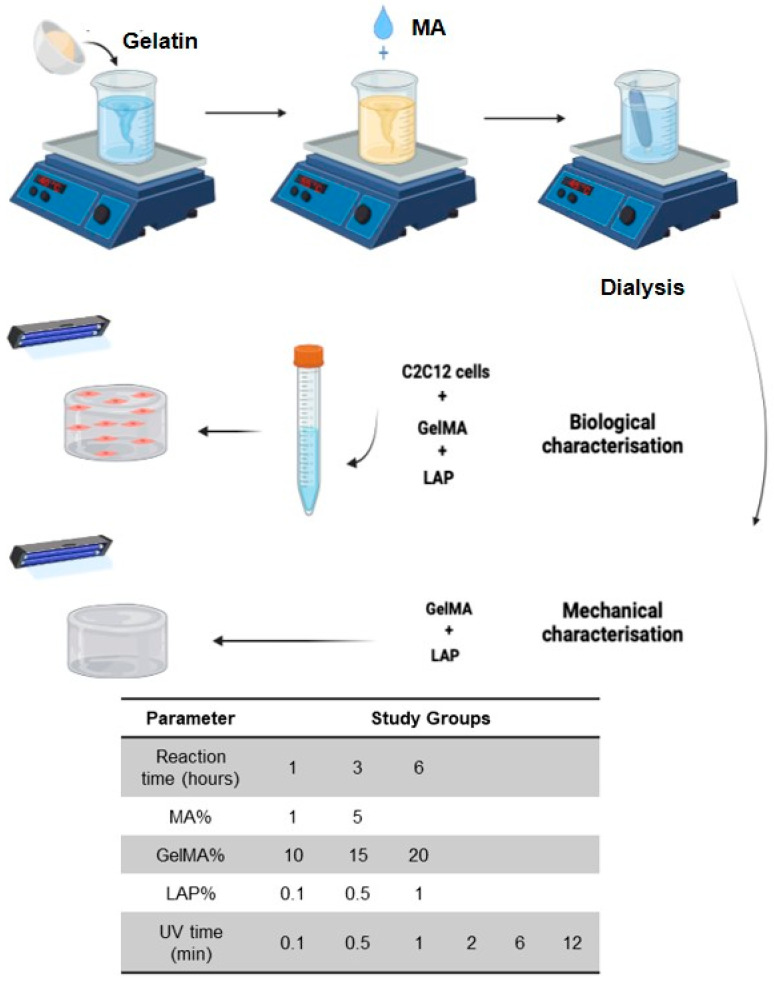
Graphical abstract showing the GelMA synthesis protocol and the experimental groups. BioRender.com was used to design this figure, accessed on 15 November 2022.

This study aimed to prepare and optimize GelMA hydrogels for craniofacial muscle regeneration by varying key synthesis parameters, including reaction time, methacrylic anhydride (MA) concentration, GelMA concentration, photoinitiator (LAP) concentration, and UV exposure duration. In this work, we systematically evaluate their mechanical and biological properties to identify optimal conditions for muscle tissue engineering applications (Figure 1). The null hypotheses were that reaction time, MA concentration, GelMA concentration, photoinitiator (LAP) concentration, and UV crosslinking duration had no effect on the mechanical or biological properties of GelMA [29,30].

## 2. Results and Discussion

In this study, GelMA was successfully synthesized, and key parameters were systematically optimized for applications in skeletal and craniofacial muscle tissue engineering. Our findings emphasize the importance of controlling both the reaction conditions and formulation components to tailor the physicochemical and biological properties of GelMA hydrogels.

### 2.1. Degree of Substitution by ^1^H-NMR

**Figure 2:**
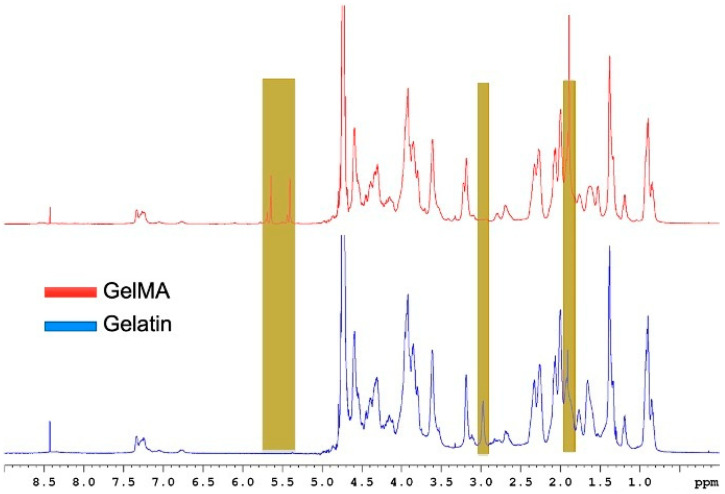
A representation of the ^1^H NMR spectra of GelMA and gelatin illustrating the peaks of interest. The peak at 5.5 ppm corresponds to the acrylic proton methacrylamide, the peak at 3 refers to the lysine methylene protons, and the peak at 1.8 represents the vinyl protons of MA.

To evaluate the effect of synthesis parameters on the degree of substitution (DS), ^1^H NMR spectroscopy was used to quantify the appearance of vinyl proton peaks (5.3–6.1 ppm), which correspond to methacryloyl groups grafted onto the gelatin. ^1^H NMR spectroscopy was performed to ensure the successful synthesis of GelMA by comparing the spectrum of GelMA with that of gelatin (Figure 2).

Figure 1 shows the ^1^H NMR spectra of GelMA and gelatin, indicating the functional groups present in GelMA. Specifically, the peak at 5.5 ppm corresponds to the acrylic proton methacrylamide, while the small peaks at 5.5 ppm adjacent to the methacrylamide represent the acrylic protons of the methacrylate groups. Additionally, the peak at 1.8, which represents the vinyl protons of MA, and the peak at 3, which refers to the lysine methylene protons, which are replaced by MA in GelMA, indicate a high degree of substitution.

**Figure 3:**
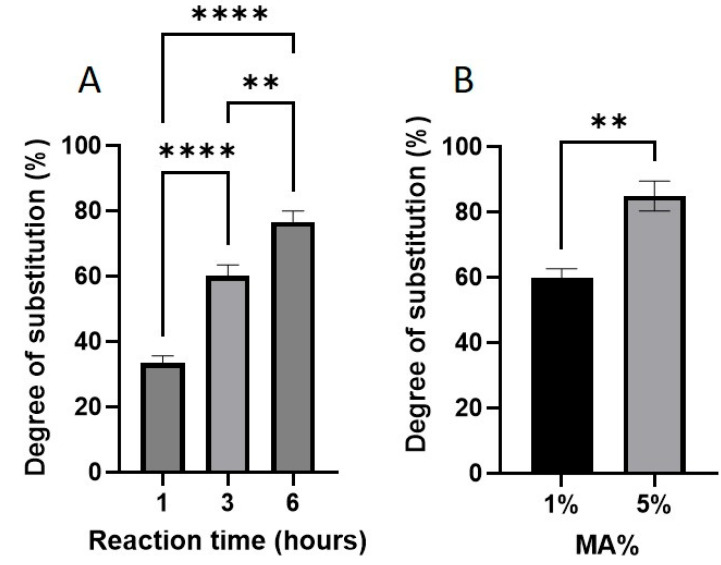
(**A**) Effect of reaction time (1, 3, and 6 h) on the degree of substitution (DS) of GelMA, calculated from ^1^H-NMR data (*n* = 3). The increase in DS with longer reaction times was statistically significant. (**B**) Effect of methacrylic anhydride (MA) concentration (1% and 5% *v*/*v*) on DS (** *p* < 0.01, **** *p* < 0.0001).

Figure 3 shows the effect of reaction time and methacrylic anhydride (MA) concentration on the degree of substitution (DS) of GelMA. As shown in Figure 3A, increasing the reaction time significantly increased the DS. After 1 h of reaction, the DS was approximately 30%, which increased to 60% after 3 h, and reached 75% after 6 h. This clearly shows that prolonged reaction times promote further methacryloylation. Figure 3B shows the effect of varying the MA content on the DS. GelMA synthesized with 1% (*v*/*v*) MA showed a DS of ~60%, whereas increasing the MA concentration to 5% resulted in a DS of ~85%—a ~45% increase.

The degree of GelMA substitution was strongly dependent on the reaction time between gelatin and methacrylic anhydride. As demonstrated by ^1^H NMR analysis, prolonging the reaction time from 1 to 6 h significantly increased the methacrylation degree from ~30% to ~80%. These findings align with those of earlier reports, such as that of [3], who observed low substitution for short reaction durations. Because DS has downstream effects on pore architecture, stiffness, and cell compatibility, careful control of the reaction time is critical for reproducibility and application-specific customization.

In addition to the reaction time, the concentration of MA used during synthesis played a pivotal role in defining the properties of the resulting hydrogel. Increasing the MA% from 1% to 5% resulted in a higher DS, which correlated with reduced swelling ratios owing to tighter crosslinking and smaller pore sizes. This observation is consistent with previous studies that reported decreased swelling with increased methacrylation [15,31].

### 2.2. MA Concentration

**Figure 4:**
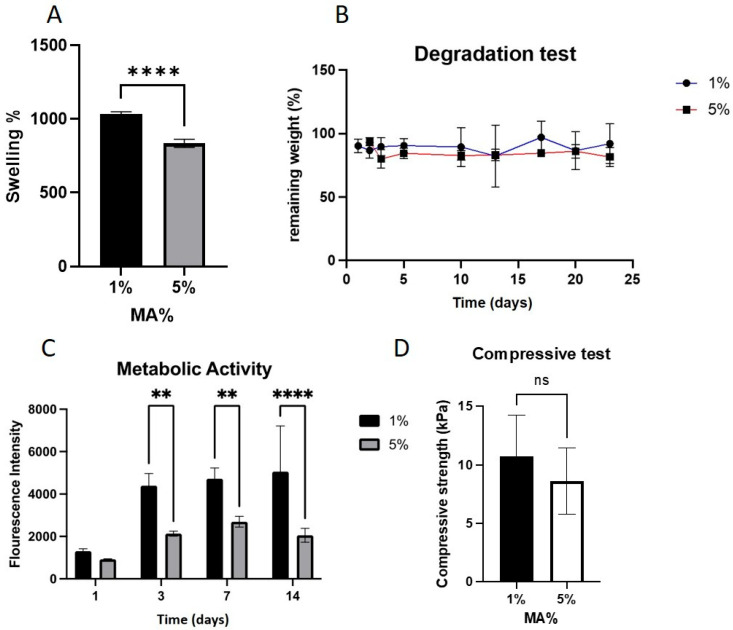
(**A**) Swelling ratio (SR) of GelMA hydrogels synthesized with 1% and 5% (*v*/*v*) methacrylic anhydride (MA), measured after 24 h in PBS at 37 °C. Increased MA content significantly reduced swelling. (**B**) Degradation profile of GelMA hydrogels synthesized with 1% and 5% (*v*/*v*) MA over 23 days in PBS at 37 °C. Both groups retained more than 80% of their original weight, indicating comparable long-term stability (*n* = 5). (**C**) Metabolic activity of C2C12 cells encapsulated within GelMA hydrogels synthesized with 1% and 5% MA over 14 days, assessed by AlamarBlue assay. The 1% MA group showed significantly higher metabolic activity on day 3. ** *p* < 0.01, **** *p* < 0.0001. (**D**) Compressive strength of GelMA hydrogels synthesized with 1% and 5% (*v*/*v*) MA at 37 °C. No significant differences were observed (*p* > 0.05). (ns = not significant).

The swelling behavior of GelMA hydrogels was assessed by immersing dried samples in PBS at 37 °C for 24 h, followed by weight analysis. Figure 4A shows the effects of MA concentration on the swelling behavior of GelMA hydrogels. Increasing the MA concentration from 1% to 5% (*v*/*v*) significantly reduced the swelling ratio (SR) of the hydrogels (*p* < 0.0001), decreasing from approximately 1000% to 800% (Figure 4A). This inverse relationship is attributed to the increased crosslinking density resulting from the higher availability of methacryloyl groups, which limits water uptake.

**Figure 5:**
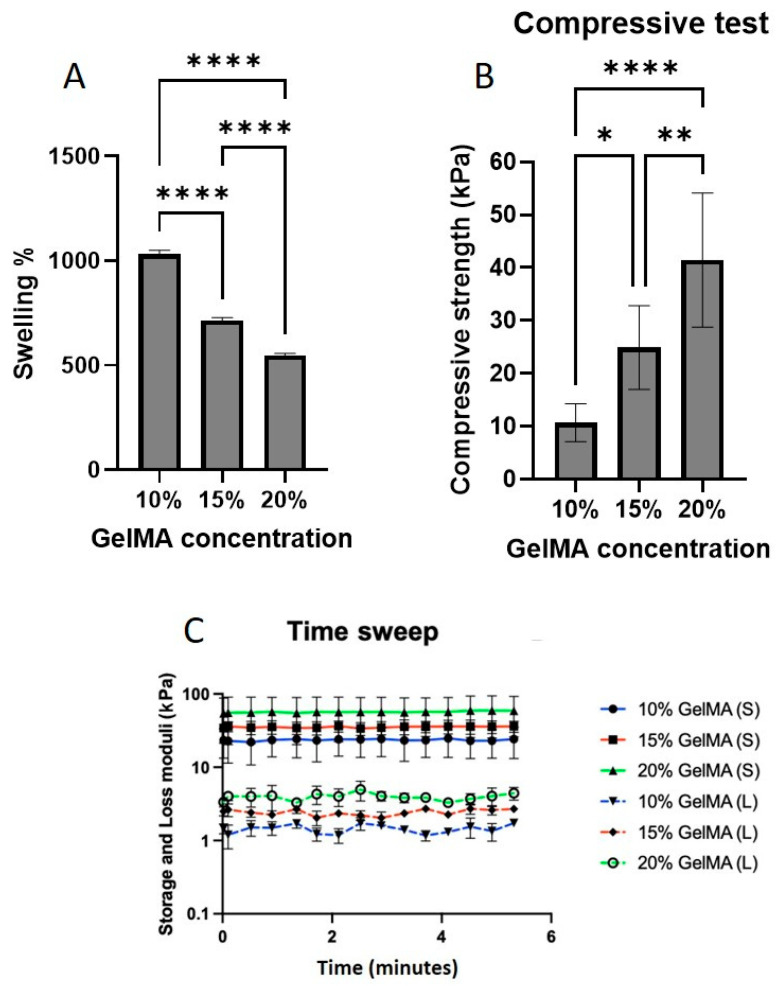
(**A**) Effect of GelMA macromer concentration (10%, 15%, and 20% *w*/*v*) on SR after 24 h in PBS at 37 °C, showing a concentration-dependent decrease. (**B**) Compressive strength of GelMA hydrogels prepared with 10%, 15%, and 20% (*w*/*v*) GelMA, showing a direct correlation between GelMA concentration and strength. (**C**) Storage and loss moduli (time sweep, 5 min) of the same hydrogels at 37 °C, with the 20% GelMA group exhibiting the highest values, indicating superior elastic response.* *p* < 0.05, ** *p* < 0.01, **** *p* < 0.0001.

The degradation behavior of GelMA hydrogels was assessed over a 23-day period by monitoring weight loss in PBS (pH 7.4) at 37 °C. Figure 4B GelMA samples prepared using 1% and 5% MA demonstrated comparable degradation profiles, retaining over 80% of their original weight after 23 days, as shown in Figure 5. This suggests that within this MA% range, the difference in the crosslink density does not significantly affect the long-term hydrolytic stability.

The cytocompatibility of the GelMA hydrogels was evaluated by measuring the metabolic activity of encapsulated C2C12 cells using the AlamarBlue assay for 14-day period. As shown in Figure 4C, hydrogels synthesized with 1% and 5% (*v*/*v*) MA exhibited low metabolic activity on day 1. However, a significant increase in metabolic activity was observed on day 3 in the 1% MA group (*p* < 0.01), whereas the 5% MA group showed only a modest increase. Both groups demonstrated relatively stable or slightly increasing metabolic activity up to day 14, although the 1% MA group consistently outperformed the 5% group.

The mechanical strength of the GelMA hydrogels was assessed at 37 °C using dynamic mechanical analysis (DMA) in the compression mode. The compressive strength of GelMA samples synthesized with either 1% or 5% MA showed no statistically significant difference, with average values of approximately 10 kPa and 8 kPa, respectively (Figure 4D). These results suggest that increasing the MA concentration within this range does not substantially affect the bulk mechanical integrity under compression.

Although the mechanical and degradation properties were not significantly affected by MA%, the biological response showed clear sensitivity. Hydrogels prepared with 1% MA supported significantly higher C2C12 metabolic activity than those with 5% MA, suggesting better cytocompatibility. The observed reduction in metabolic activity may be attributed to the increased replacement of bioactive gelatin proteins with synthetic methacrylate groups and possibly the incomplete purification of unreacted MA, which can be cytotoxic. This work emphasizes that reducing the methacrylic anhydride concentration from 5% to 1% significantly decreased cytotoxicity while maintaining mechanical strength, underscoring the role of controlled methacrylation in enhancing cell compatibility.

### 2.3. GelMA and LAP Concentrations

To assess the swelling behavior, varying the GelMA macromer concentration from 10% to 20% (*w*/*v*) produced a concentration-dependent reduction in SR (Figure 5A). Specifically, the SR decreased from 1000% (10%) to 700% (15%) and then to 500% (20%) (*p* < 0.0001), highlighting the impact of the polymer content on the network density and porosity.

GelMA concentration had a pronounced effect on the mechanical behavior. As shown in Figure 5B, increasing the GelMA content from 10% to 20% led to a significant increase in compressive strength from 10 kPa to 40 kPa (*p* < 0.0001). Corresponding oscillation time sweep tests demonstrated a strong positive correlation between GelMA concentration and both storage and loss moduli, with the 20% GelMA group showing the highest values (Figure 5C), suggesting enhanced elasticity. Notably, hydrogels containing ≥0.5% LAP could not be reliably measured because of structural instability and rapid disintegration, likely due to over-polymerization or network defects from high radical flux.

**Figure 6:**
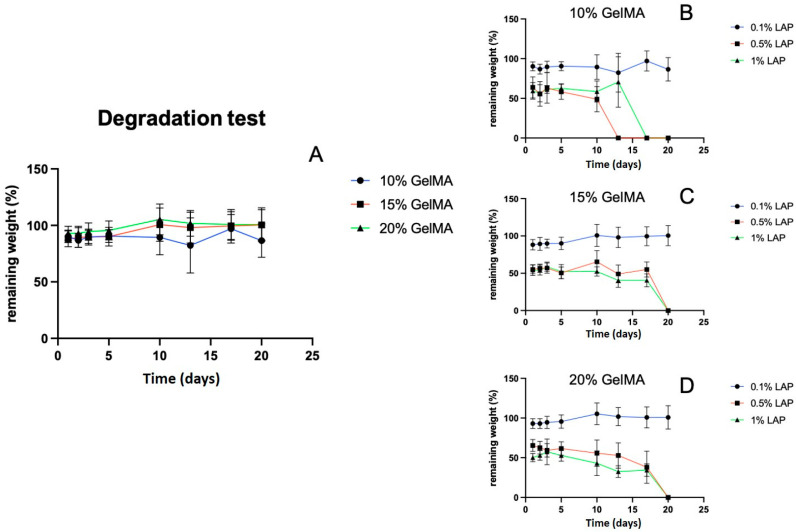
(**A**) Effect of GelMA concentration on hydrogel degradation. (**B**–**D**) Effect of LAP concentrations (0.1%, 0.5%, and 1%) on the degradation of 10%, 15%, and 20% GelMA hydrogels, respectively. Higher LAP concentrations resulted in faster degradation, with complete structural loss occurring between days 16–20 depending on GelMA content (*n* = 5).

The degradation behavior of hydrogels fabricated with varying GelMA (10%, 15%, 20%) and LAP (0.1%, 0.5%, 1%) concentrations revealed a strong dependence on LAP content. Although GelMA% alone did not significantly affect degradation over 23 days (Figure 6A), increasing the LAP concentration to 0.5% or 1% resulted in rapid initial degradation, with 40–50% mass loss observed after 24 h (Figure 6B–D). Complete degradation occurred by day 16 in the 10% GelMA group and by day 20 in the 15% and 20% GelMA groups, respectively. This behavior is likely due to excessive free radicals during crosslinking, which may cause network defects or partial chain scission.

**Figure 7:**
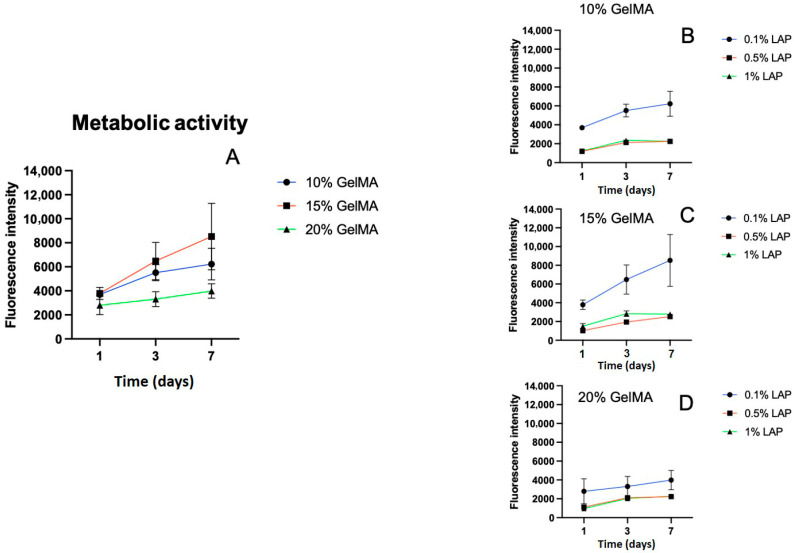
(**A**) Metabolic activity of C2C12 cells in GelMA hydrogels with varying polymer concentrations (10%, 15%, and 20%) over 7 days, although 10% and 15% GelMA showed higher metabolic activity, they were not statistically significant. (**B**–**D**) Effect of LAP concentrations (0.1%, 0.5%, 1%) on C2C12 metabolic activity in 10%, 15%, and 20% GelMA hydrogels, respectively, over 7 days. The 20% GelMA group consistently showed the lowest metabolic activity, while 0.1% LAP yielded the highest activity across all groups.

Varying the GelMA content also impacted the cellular behavior. As illustrated in Figure 7A, hydrogels made from 20% GelMA showed the lowest metabolic activity across the first 7 days. In contrast, 10% and 15% GelMA formulations supported higher and more stable metabolic responses, with the 15% group showing a mild increase on day 7.

LAP concentration strongly influenced cell metabolic activity. Hydrogels photopolymerized with 0.5% or 1% LAP showed a marked decrease in metabolic activity compared to those photopolymerized with 0.1% LAP (Figure 7B–D), indicating potential cytotoxic effects at higher photoinitiator concentrations.

The GelMA polymer concentration also had a pronounced impact on hydrogel behavior. Higher GelMA concentrations (20%) resulted in lower swelling ratios and reduced metabolic activity, while enhancing mechanical strength. These findings are consistent with published data showing that increasing the GelMA content densifies the network, reduces the pore size, and improves the load-bearing capacity [15,16]. However, such densification restricts cell proliferation and nutrient transport, leading to reduced viability. Increasing the GelMA polymer concentration from 10% to 20% improved stiffness but led to reduced metabolic activity at the highest concentration, likely due to limited nutrient and oxygen diffusion in denser matrices. Interestingly, degradation rates up to 23 days were not significantly influenced by GelMA concentration, suggesting that other factors, such as enzyme accessibility or photoinitiator concentration, may dominate degradation kinetics in this context.

This study demonstrates that the amount of methacrylic anhydride (MA%) added during synthesis of GelMA had more statistically significant influence on cellular responses than on the mechanical properties of GelMA. Increasing MA% to 5% resulted in a marked reduction in metabolic activity, indicating increased cytotoxicity, whereas maintaining MA% at 1% or below preserved cell viability. In contrast, varying GelMA concentration (10–20%) primarily affected mechanical performance, with higher concentrations improving stiffness and stability but slightly reducing metabolic activity at 20%. Collectively, these findings emphasize the importance of maintaining low MA% (≈1%) while optimizing GelMA concentration to achieve the desired balance between mechanical integrity and cytocompatibility.

The photoinitiator (LAP) concentration is another critical factor in photocrosslinkable systems. While higher LAP concentrations (0.5% and 1%) promoted rapid crosslinking, they also induced severe cytotoxicity and poor hydrogel integrity. The metabolic activity of encapsulated cells was drastically reduced at higher LAP%, and mechanical testing was not feasible because of sample disintegration. In contrast, 0.1% LAP yielded intact hydrogels with relatively high metabolic activity and measurable mechanical strength measurements. These results suggest that minimizing LAP concentration helps preserve scaffold structure and biocompatibility, likely by avoiding excessive free radical production that can damage both the polymer and cells. Importantly, this work is the first to systematically evaluate the influence of LAP concentrations (0.1%, 0.5%, and 1%) on GelMA hydrogel properties. Higher LAP levels (≥0.5%) increased cytotoxicity and accelerated degradation, and these samples failed to form stable, crosslinked networks, indicating excessive radical generation and incomplete polymerization at higher initiator concentrations.

### 2.4. UV Exposure Time

**Figure 8:**
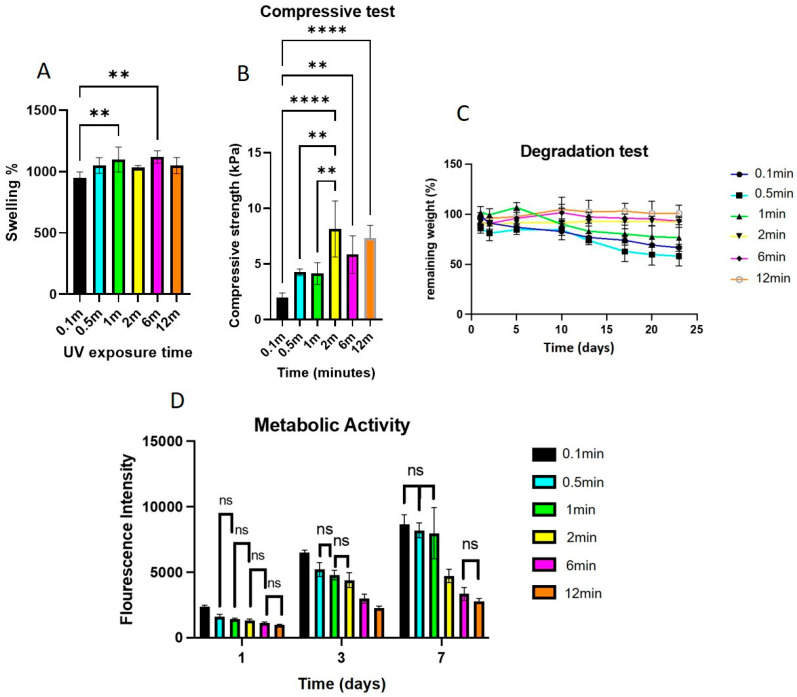
(**A**) Influence of UV exposure times of 0.1 to 12 min on the swelling ratio (SR) of GelMA hydrogels. Samples cured for 10 s showed significantly lower SR compared to longer exposure times, with no significant differences observed among the other groups (** *p* < 0.01). (**B**) Compressive strength of GelMA hydrogels cured under UV for 0.1 to 12 min. UV exposure for ≥2 min significantly enhanced mechanical strength compared to shorter times. ** *p* < 0.01, **** *p* < 0.0001. (**C**) Influence of UV exposure time (0.1, 0.5, 1, 2, 6, and 12 min) on the degradation profile of GelMA hydrogels over 23 days in PBS at 37 °C. Hydrogels exposed for ≤1 min degraded faster (30–40% weight loss), which was statistically significant compared to the other groups (*p* < 0.05), illustrating improved hydrogel stability with increased exposure. *n* = 5. (**D**) Influence of UV exposure time (0.1–12 min) on the metabolic activity of encapsulated C2C12 cells in GelMA hydrogels. An inverse relationship was observed, with longer UV exposure correlating with decreased cell metabolic activity. While some comparisons were not significant (ns), all other differences were statistically significant (*p* < 0.05).

The UV crosslinking time was optimized. Figure 8A shows that GelMA samples crosslinked for only 10 s exhibited significantly lower SR than those crosslinked for 30 s or longer. However, no statistically significant differences in SR were observed among samples exposed for 30, 60, or 120 s, suggesting that a threshold for complete crosslinking was achieved within the first 30 s under the given conditions.

The UV exposure time also affects the mechanical performance. As shown in Figure 8B, GelMA exposed to UV light for ≥2 min exhibited significantly higher compressive strength than that crosslinked for 1 min or less (*p* < 0.01). This finding reinforces the importance of adequate curing duration for network formation and structural integrity.

The UV exposure time had a pronounced impact on the long-term hydrogel stability. As shown in Figure 8C, samples crosslinked for ≤1 min exhibited significant degradation, losing 30–40% of their initial weight. However, increasing the UV exposure time to 2 min or more significantly improved hydrogel stability (*p* < 0.01), with minimal additional degradation observed over the 23-day period. This supports the idea that an adequate crosslinking time is critical for forming a stable network.

Furthermore, as shown in Figure 8D, longer UV exposure times resulted in a reduction in metabolic activity. A clear inverse correlation was observed between UV curing duration and cell metabolic activity, with samples exposed to ≥2 min of UV light showing significantly lower metabolic activity (*p* < 0.05) than those cured for ≤1 min.

The UV exposure time required to initiate crosslinking also influenced the dual outcomes of mechanical strength and cell metabolic activity. Increasing UV exposure from 10 s to 2 min enhanced compressive strength, but exposure beyond 2 min had minimal additional effect. The metabolic activity of the encapsulated C2C12 cells was inversely correlated with UV duration. Prolonged irradiation beyond 2 min (particularly 6 min and 12 min) caused a sharp decline in metabolic activity, suggesting photoinitiator- cytotoxic effects. Based on these results, a UV exposure of 1–2 min was identified as the optimal compromise between mechanical robustness and cellular viability. Although the cells were not completely nonviable even after 12 min of exposure, shorter UV exposure durations were clearly more favorable. These findings reinforce the notion that, while extended crosslinking times can optimize structural integrity, a trade-off with cell health must be considered, particularly in applications involving live cell encapsulation.

Recent studies have demonstrated that GelMA hydrogels provide a highly tunable and biologically favorable matrix for skeletal muscle engineering. For example, a study showed that GelMA concentrations from 5% to 15% significantly influenced C2C12 spheroid sprouting, mechanical stiffness and myogenic differentiation [4]. Another study concluded that nano-engineered GelMA scaffolds with controlled IGF-1 release improved proliferation and differentiation of muscle progenitors [29].

Overall, this data suggests that synthesis and processing parameters, such as MA%, GelMA%, LAP%, and UV exposure time, must be carefully optimized to balance structural functionality with cytocompatibility. For craniofacial muscle regeneration applications, where both mechanical resilience and cell viability are required, a formulation comprising 1% MA, 10–15% GelMA, 0.1% LAP, and a UV exposure time of 1–2 min appears to provide an optimal compromise. These findings provide valuable insights for the rational design of GelMA hydrogels tailored for muscle tissue engineering applications.

This study was performed under controlled in vitro conditions, which may limit the direct extrapolation of the results to in vivo environments. Further research is needed to evaluate the long-term stability and biocompatibility of GelMA hydrogels in physiological settings. Nevertheless, the findings offer valuable insights into optimizing the synthesis parameters to achieve a balance between mechanical integrity and cytocompatibility, thereby advancing the development of GelMA-based biomaterials for craniofacial and skeletal muscle regeneration.

## 3. Conclusions

This study systematically examined key synthesis and processing parameters influencing the physicochemical and biological properties of GelMA hydrogels for craniofacial and skeletal muscle regeneration. Methacrylic Anhydride (MA) concentration and reaction time significantly impacted the degree of substitution (DS), water uptake, and cell metabolic activity, where higher values increased DS but reduced biological performance. Likewise, higher GelMA concentrations (10–20%) enhanced mechanical strength but limited water uptake and metabolic activity. Additionally, LAP concentrations ≥ 0.5% and prolonged UV exposure reduced metabolic activity, while conditions with 0.1% LAP and 1–2 min of UV exposure provided the best balance between mechanical integrity and biological compatibility. These findings provide a practical framework for optimizing GelMA hydrogel fabrication to improve reproducibility and support application-specific tissue engineering strategies.

Building on the present findings, future studies will focus on validating the optimized GelMA formulations in in vivo models of skeletal muscle regeneration and comparing their mechanical performance with native muscle tissue. The optimized synthesis parameters identified in this work, particularly 1% methacrylic anhydride concentration, 10–15% GelMA, 0.1% LAP, and 1–2 min UV exposure, provide a promising foundation for developing cytocompatible yet mechanically stable constructs. Further work will explore fine-tuning crosslinking conditions to achieve higher strength without compromising metabolic activity, and to improve long-term degradation behavior under physiological conditions. Integration of these optimized GelMA hydrogels into 3D bioprinted and multilayer scaffold systems will be pursued to enhance tissue alignment, nutrient diffusion, and vascularization. These advancements will help establish standardized, reproducible GelMA-based platforms for skeletal muscle and other soft tissue regeneration applications.

## 4. Materials and Methods

### 4.1. GelMA Synthesis

GelMA was synthesized following the protocol detailed in [3]. Briefly, Gelatin from bovine skin (225 g) was dissolved in Phosphate-Buffered Saline (PBS) at 10% *w*/*v* at 50 °C using a glass round-bottom beaker inside a water bath to ensure uniform heating. Methacrylic anhydride (MA) was added dropwise at various concentrations (1% and 5% *v*/*v*) while maintaining the temperature at 50 °C and stirring speed at approximately 700 rpm using a magnetic stirrer (Stuart US152 instrument Cole-Parmer, Cambridgeshire, UK). After a defined reaction time (1 h, 3 h, or 6 h), the reaction was terminated by diluting and neutralizing the pH of the solution by adding 5-fold of warm PBS (50 °C). The solution was dialyzed against warm distilled water (12–14 kDa MWCO) for 5 days. Then, the solution was placed in airtight containers in a –80 °C freezer for 24 h before it was lyophilized using Alpha 1-2 LDplus lyophiliser from Martin Christ (SciQuip Ltd., Newtown Wem Shropshire, UK) until a dry white GelMA macromer was obtained (~72 h). All materials and consumables were purchased from Sigma-Aldrich (Merck Life Sciences UK Ltd., Poole, Dorset, UK) unless otherwise specified.

### 4.2. Proton-Nuclear Magnetic Resonance Characterization

Proton-Nuclear Magnetic Resonance (^1^H NMR) was performed in D_2_O to evaluate the incorporation of methacryloyl groups and confirm reaction progress at various time points. 20 mg of GelMA or gelatin was dissolved in D_2_O at 50 °C, then transferred into NMR tubes for analysis. NMR spectra were acquired using a Bruker Avance Neo 700 MHz spectrometer (Bruker Ltd., Coventry, UK) at room temperature. All spectra underwent phase and baseline correction prior to the integration of the signals of interest. Specifically, the peak at approximately 3 ppm, which corresponds to the methylene protons of lysine, was used for analysis (processed with TopSpin software (4.1.1), Bruker Ltd., Coventry, UK). The chemical shift scale was adjusted to account for the residual solvent signal of D_2_O (^1^H = 4.79 ppm) [28]. The degree of substitution (DS) was determined using Equation (1) [32].

(1) $$DS(\%)=\left(1-\frac{lysine\;methylene\;proton\;of\;GelMA}{lysine\;methylene\;proton\;of\;Gelatin}\right)\times 100$$

### 4.3. Hydrogel Preparation and UV Crosslinking

Lyophilized GelMA was dissolved in PBS to reach 10%, 15%, or 20% concentrations (*w*/*v*), combined with 0.1%, 0.5%, or 1% LAP (*w*/*v*), and crosslinked by exposure to 405 nm UV light for 10 s, 30 s, 60 s, 2 m, 6 m or 12m in cylindrical molds (96-well plates).

### 4.4. Swelling Test

Cylinder-shaped hydrogel samples (diameter 6 mm, thickness 2 mm) were prepared, crosslinked and lyophilized using the above mentioned protocol (*n* = 5). The freeze-dried samples were weighed dry (Wd) using an Ohaus balance (OHAUS Europe GmbH, Nänikon, Switzerland), then immersed in PBS at 37 °C for 24 h at static conditions. Then, samples were removed using small spatula and carefully dabbed using blue laboratory tissue to removed excess water. Finally, samples were reweighed (Ws). The swelling ratio was calculated as:

(2) $$SR=\frac{\left(Ws-Wd\right)}{Wd}\times 100$$

### 4.5. Mechanical Testing

Compression tests were conducted using a Discovery Dynamic Mechanical Analyser DMA 850 (TA Instruments, Elstree, Hertfordshire, UK). Cylinder-shaped samples (6 mm diameter, 2 mm height) were compressed at 0.5 mm/min at 37 °C with a soaking time of three minutes, and compressive modulus was calculated from the initial linear region. Additionally, a DMA was employed to perform an oscillation time sweep at 37 °C for 5 min, utilizing a frequency of 1 Hz and a strain of 0.1%, to assess the viscoelastic characteristics of the crosslinked hydrogels.

### 4.6. Degradation in PBS

Hydrogels were crosslinked, weighed (W_0_), and incubated in PBS at 37 °C. Every 2 days, samples were removed, blotted, and weighed (Wt). The remaining mass was calculated as:

(3) $$\mathrm{Remaining}\;\mathrm{weight}\;(\%)\;=100+\frac{\left(W_{t}-W_{0}\right)}{W_{0}}\times 100$$

### 4.7. Gel Sterilization and 3D Cell Culture

The GelMA hydrogel solution was prepared following the established protocol mentioned earlier and sterilized by filtration through a 0.22 μm syringe filter [33,34]. A volume of 100 μL of the sterile GelMA solution was dispensed into each well of a 96-well plate. Subsequently, 2.5 × 10^4^ C2C12 cells were added to each well, and the cell-hydrogel mixture was gently mixed using a micropipette tip to prevent bubble formation. The samples were then crosslinked via UV irradiation before being transferred to a cell culture incubator maintained at 37 °C with 5% CO_2_ for further incubation.

### 4.8. Cell Metabolic Activity

The metabolic activity of cells was assessed using the AlamarBlue assay. In this assay, the non-fluorescent dye resazurin is reduced to the fluorescent compound resorufin by cellular metabolic enzymes. Thus, the measured fluorescence intensity is proportional to the number of metabolically active cells. A 10% AlamarBlue (AB) solution (Fisher Scientific UK Ltd., Loughborough, UK). was prepared by diluting the AB reagent in complete growth medium at a 1:9 ratio. A volume of 100 μL of the AB solution was added to each well of the 96-well plate containing the samples. The plate was then incubated at 37 °C for 4 h on an orbital shaker (Stuart Mini Orbital Shaker SSM1, Cole-Palmer, Cambridgeshire, UK) to ensure uniform reagent distribution and facilitate diffusion into the hydrogel samples.

Following incubation, 100 μL of the AB solution from each well was transferred to a black 96-well plate with a clear bottom. Fluorescence intensity was measured using a Biotek FLx800 microplate reader (Biotek UK, Swindon, UK) at excitation/emission wavelengths of 560 nm and 590 nm, respectively.

### 4.9. Statistical Analysis

The data were analyzed using appropriate statistical tests based on the experimental design, including independent *t*-tests, one-way ANOVA and two-way ANOVA followed by Tukey’s post hoc test (GraphPad Prism 9, San Diego, CA, USA). A *p*-value < 0.05 was considered statistically significant.

## Data Availability

Data will be made available upon request to the corresponding author.

## Abbreviations

The following abbreviations are used in this manuscript:

GelMA	Gelatin Methacryloyl
MA	Methacrylic Anhydride
DS	Degree of Substitution
PBS	Phosphate-Buffered Saline
LAP	lithium phenyl-2 4 6-trimethyl-benzoyl phosphinate
UV	Ultraviolet
^1^H NMR	Proton Nuclear Magnetic Resonance
